# Promoting Active Learning and Student Engagement in Two Different Graduate Courses for Veterinary and Animal Sciences: Cases From Mexico and Denmark

**DOI:** 10.3389/fvets.2022.822409

**Published:** 2022-01-28

**Authors:** Rocio Angélica Ruiz-Romero, Einar Vargas-Bello-Pérez

**Affiliations:** ^1^Departamento de Medicina y Zootecnia de Rumiantes, Facultad de Medicina Veterinaria y Zootecnia, Universidad Nacional Autónoma de México, Ciudad de México, Mexico; ^2^Department of Veterinary and Animal Sciences, Faculty of Health and Medical Sciences, University of Copenhagen, Copenhagen, Denmark

**Keywords:** animal science, education, veterinary, Scandinavia, Latin America, microbiology, physiology

## Introduction

There are many criticisms that have arisen over the years due to the use of animals for teaching in various fields of study such as physiology, which is why several alternatives have been implemented and have increased in some pedagogical approaches. Veterinary teachers and administrators are rarely credited with being committed for developing effective curricular methods that allow reductions in the use of animals (1). For example, anatomy knowledge from small (i.e., dogs and cats) and large animal species (i.e., horses and cattle), and experience associated to physiological responses are required for students related to veterinary and animal sciences. These students are expected to have basic handling skills and know the procedures for performing intubation, venipuncture, giving injections, and catheter placement before they can forward with more complex procedures (1).

The challenge of integrating new learning prototypes into previously existing curricula includes dealing with students who may be disappointed by losing physical contact with animals, so new ways must be discovered to offer experience in live animal surgery (1, 2). Today's tendency is that more and more students disapprove invasive animal handling procedures. By forcing students to handle animals in ways that can be considered painful, damaging, stressful, and even lethal, various reactions can occur (3). Psychological outcomes for students in combination with educational, scientific, environmental, and social outcomes are a compelling reason for replacing the use of animals for educational purposes (3).

When teaching animal physiology, practical demonstrations, using laboratory animals, is very common and useful to exemplify and strengthen theoretical concepts (4). When working with laboratory animals, students will be able to acquire greater ability and improve analytical thinking, which is consistent with the didactics of problem-based learning (5). In medical education, laboratory learning as a dynamic element to improve practical skills helps to fulfill learning objectives (6, 7). In addition, data obtained through laboratory practices help teachers to propose specific questions, encouraging students to seek answers that support their hypotheses and problem solving (7).

There are several investigations reporting that students who use virtual laboratories that have been created to provide alternatives in the practices of physiology can learn and achieve the same level of knowledge or even better than those students who have used live animals (7, 8). Alternative methods also have a number of advantages (9, 10) such as:

1) Decrease in teaching time as well as the costs involved
2) Better understanding of the consequences of drugs with the probability of making fewer mistakes during experimentation
3) Explanation of complex procedures using electronic devices
4) Individual analysis and analysis of students in their own time
5) Can be applied to audiovisual and kinesthetic learners
6) Indefinite repetition of the experiments.

However, some disadvantages of alternative learning methods have been identified, and include the lack of appropriate devices, training time for the demonstrator, skills, and above all, they decrease social interaction and motivation, little or no face-to-face interaction (5, 7).

In our institutions, both in Mexico and in Denmark, the vast majority of these advantages have been verified and experienced, which is why it has also contributed to motivating other teachers to substitute the use of animals for other alternatives.

Medical microbiology courses are usually intensive courses that require a great deal of commitment from the students and can be difficult to teach in a way that benefits all of them, the curricula that students should take goes beyond memorization and expect students to analyze and synthesize the new knowledge acquired (11). A report prepared by an educational working group of the American Society for Microbiology identified general concepts in microbiology that students should understand like evolution of microorganisms such as bacteria, as well as their structure and their role as virulence factors, metabolism, as well as the impact of these microorganisms on public health (11, 12).

With these approaches, major changes have been made in the curriculum within the field of microbiology, with the purpose of improving the understanding of the theoretical part by students, their daily work and throughout the semester and the containment of knowledge of microbiology beyond the time required in class (11).

In some American and European universities, one of the main concerns when revising microbiology courses are stop memorizing and promote critical thinking, improve laboratory skills, keep constantly updated to new knowledge related to microbiology and that revised contents are outstanding so that students maintain interest in this area of Medicine (12).

Typically, microbiology courses help students to enter a great biological diversity, so that a typical course only covers a small population of microorganisms (12). One challenge that teachers face in any natural science course is teaching in a way that helps students to create superior cognitive skills. This challenge can be especially alarming in teaching microbiology due to the large number of scientific findings that are discovered every day (12, 13).

When teaching microbiology, laboratory practices are essential since students learn in an environment where they acquire practical skills that they cannot acquire from reading. For a microbiology course to be complete, teachers must evaluate the practical part that is done in teaching laboratories. In this regard, over the last 20 years there have been drastic changes in the way of teaching that is given in laboratories ensuring quality in education (14).

Normally, laboratory classes are structured to follow protocols that simply serve as validation routines of some concepts that are covered in theoretical classes (15).

Advances in technology have favored the expansion of courses, these can combine face-to-face practices within the laboratory with online learning with the so-called “virtual laboratories,” these types of laboratories are attractive for different faculties since they can satisfy the needs of the students and of the same faculty since the number of physical laboratories can be reduced or even eliminated. The changes that can be seen within the teaching of microbiology have a positive impact on the perception of students about learning within the laboratory (13, 14, 16).

Physical teaching laboratories provide students with the opportunity to learn in a practical environment with their peers and with an instructor. It has been reported that students feel more comfortable receiving direct feedback from the teacher rather than receiving feedback through some electronic media. This is due the fact that students feel more responsibility when working with laboratory samples, increasing interest for laboratory work (13, 14). Interpersonal relationships between students increase motivation for the course and by receiving feedback face-to-face with their instructor they can obtain some extra learning experiences. However, due to the high costs generated by physical laboratories that includes material, infrastructure and hiring of teaching staff, some universities have a small number of these types of laboratories (14).

By using simulators in hands-on teaching, students can access labs from anywhere, eliminating construction and material costs, and online exercises give students the chance to practice the exercises multiple times, and thus which turns out to be an extremely expensive activity in physical laboratories. Another study found that being able to perform the exercises a large number of times was well-received by students, further supporting the profitability of this type of laboratory (14). However, its cost is related to the educational level and the course work area and in this regard, higher education levels may require more expensive simulators (13, 14).

The efficiency of online learning is equivalent to or greater than that achieved in in-person learning. Some investigations have reported that students performed similarly in-person laboratories and virtual; in addition to that, there are some extra benefits when using virtual laboratories such as shorter class time, less hiring of teachers and greater students' motivation. Therefore, learning that combines physical and online resources closes the gap between financial limitations that may exist in some universities and the preferences or learning requirements of students, different studies have found that when assigning tasks online before the activities of the Physics lab enhance students' confidence and motivation to learn (13–16). Students who used a physical laboratory and a virtual laboratory experienced increases in both knowledge and self-efficacy in microbiology (14, 16).

The objective of the present opinion is to highlight examples of veterinary and animal sciences courses that have developed alternative teaching resources and methods, while maintaining traditional educational approaches.

## Mexico

The first case to discuss is from the Faculty of Veterinary Medicine and Animal Science (FMVZ) from The National Autonomous University of Mexico (in Spanish: Universidad Nacional Autónoma de México, abbreviated as UNAM) is the oldest in North America and the largest in Latin America, it was founded in 1551. It is a member of the American Association of Colleges of Veterinary Medicine (AAVMC) and is fully accredited by The American Veterinary Medical Association Council on Education (AVMA COE) (17).

The FMVZ offers Master's program in Production Sciences and Animal and in Veterinary Medicine and Animal Science in both programs, veterinarians and students from related careers such as Biology, Animal Science and Agronomy are accepted. In the two-master's programs, the course “Microbiology and Immunological Aspects of Mammary Gland in Domestic Ruminants” is offered as an elective course. The aim of this course is to analyze the main defense mechanisms of the mammary gland against bacteria that are frequently associated in cases of mastitis, as well as the routes used to eliminate the infectious agent, through the reinforcement of theoretical concepts, discussion of research articles and laboratory practices, to improve the quality and quantity of milk produced.

This course includes some laboratory demonstrations, and it is divided by theoretical lectures and at the end of each topic, seminars/discussions of research articles are held on expression of some immune response factors during mastitis, as well as discussion of some clinical cases of mastitis correlating the clinical signs of the animal with the laboratory diagnosis and metabolic characteristics of bacteria responsible for mastitis that are detected in the laboratory.

## Denmark

The second case for discussion is from the Department of Veterinary and Animal Sciences of the Faculty of Health and Medical Sciences, from the University of Copenhagen, which is among the 7 best universities in the world in Veterinary Science according to the QS World University Rankings 2021^1^ Bachelor and master's programs for veterinary medicine are taught in Danish and for the animal science program, bachelor is taught in Danish and master's in English (18).

In this case, graduate students from veterinary and animal sciences are taught theoretical and practical activities for a course called “Experimental Animal Nutrition and Physiology.” The course is divided by practical demonstrations, readings, exercises, and seminars. Students work in groups through the course. This course has a thematic content that covers some experimental techniques in order to evaluate animal function and the metabolism of nutrients at the animal and organ level (*in vivo/in vitro*). This encompasses a theoretical introduction and practical explanations of key techniques. A 30-h block that meets the educational requirements of the EU directive 2010/63/EU (function “a” and “d”) and the Danish National Authority *Dyreforsøgstilsynet* for those who handle experimental animals or collaborate in experiments with animals. A diploma is issued after passing the course. The necessary logistics behind *in vivo* or *in vitro* studies, as well as to critically estimate the results that are achieved from those studies is also discussed using some published papers related to the specific weekly topic. This includes criticism on protocol planning, experimental design, and data analysis. In this course, the evaluation that is carried out through a written exam is mandatory to obtain a diploma that is approved by the EU and be authorized to work with experimental animals. In order to pass the course, each student must euthanize laboratory rodents. The course includes theoretical lectures, some laboratory demonstrations using simulators (i.e., blood samplings and venipuncture) and twice a week seminar. The course includes practical exercises on live animals. In addition, students have seminar discussions of handout papers, emphasis is placed on the critical appraisal of experimental designs, techniques and approaches in relation to the goals and ethics on animal use, as well as the results and conclusions obtained. The course evaluation prototype is based on a survey-based model.

## Discussion On Learning Outcomes From The Cases of Mexico and Denmark

Table 1 shows a detailed description of learning outcomes based on knowledge, skills, and competences of the courses Microbiology and Immunological Aspects of Mammary Gland in Domestic Ruminants (Mexico) and Experimental Animal Nutrition and Physiology (Denmark).

**Table 1:** Learning outcomes based on knowledge, skills, and competences of the courses Microbiology and Immunological Aspects of Mammary Gland in Domestic Ruminants (Mexico) and Experimental Animal Nutrition and Physiology (Denmark).

			**Learning outcomes**		
**Course**	**Country**	**University**	**Knowledge**	**Skills**	**Competences**
Microbiology and Immunological Aspects of Mammary Gland in Domestic Ruminants	Mexico	Faculty of Veterinary Medicine and Animal Science, National Autonomous University of Mexico	• Identify the anatomical structures of the mammary gland and the differences that exist between cattle, sheep, and goats. • Identify the defense mechanisms of the mammary gland, from anatomical barriers, cellular defense systems, and soluble defense factors. • Associate the different mechanisms of bacterial recognition and interaction of the innate and adaptive immune response, from the recognition of the pathogen, the inflammatory process and the cellular and humoral immune response involved in the resolution of inflammation. • Identify the bacterial morphology of Gram-positive and Gram-negative bacteria. • Describe the invasiveness mechanisms and virulence factors of *Staphylococcus* spp. as an example of Gram-positive bacteria and *E. coli* as an example of Gram-negative bacteria. • Associate the evasion of the immune response by the pathogen as part of the pathogenicity mechanisms of bacteria	• Ability to describe the main anatomical differences between cattle, sheep and goats. • Ability to identify which are the anatomical defense mechanisms that prevent bacterial invasiveness. • Ability to describe the physical and chemical changes in milk as a consequence of the inflammatory process. • Ability to identify the suspected bacterial agent based on changes in the mammary gland and milk. • Ability to identify the most common pathogenicity mechanisms using traditional bacteriological tests. • Ability to explain some causes for which the mammary gland is unable of eliminating the bacterial agent.	• Ability to interact with veterinarians responsible for milk production units to improve mastitis prevention strategies. • Ability to understand the importance of early diagnosis of the disease and proper treatment
Experimental Animal Nutrition and Physiology	Denmark	Faculty of Health and Medical Sciences, University of Copenhagen	• Define the elemental principles behind empirical methods for measuring nutritional characteristics of feed intake, feedstuffs, chewing activity, *in vivo*, and *in vitro* digestibility. • Define the methods for measuring digestibility in different segments of the digestive tract in ruminant and monogastic animals by use of cannulation and marker techniques, passage rate and digestion kinetics. • Define the intellectual background of energy transfer from cellular to the whole-body level, techniques and methods for measuring nitrogen, energy balances, heat production and substrate oxidation. • Define the value of molecular biological procedures in analysis of cell and tissue function. • Define catheter procedures used to study tissue and organ nutrient fluxes, practical guidelines for sampling of blood, rumen fluid etc. • Define convenient empirical designs and basic statistical approaches for use with these methodologies. • Define the legislation, supervision and management of laboratory animals. • Assimilate the conclusion from the methodologies, empirical design work and legislative aspects to obtain an understanding of the ethical use of animals in research	• Ability to define principles and methods used in selected *in vivo* manifestations. • Ability to figure out experimental results/data from different *in vivo* measurements. • Ability to debate limitations, advantages, and their potential utilization of the different empirical methods. • Ability to illustrate empirical results based on intellectual knowledge. • Ability to employ relevant legislation for conducting animal experiments in an ethical way. • That the student exhibits the capacity to euthanize laboratory rodents.	• Be able to collaborate with other proffesors, both inter and intra disciplinary at different levels and with different degree of authority. • Be able to embrace above-described learning and skills. • Be able to propagate methodology and obtained results to specialists, lay persons and general public. • Be able to execute animal experiments legally according to EU directive 2010/63/EU function “a” and “d.”

In both courses, a theoretical-practical teaching is required for students to develop skills necessary to solve problems within their professional career. Some practices carried out face-to-face can become too expensive and the student needs to practice several times to master some techniques. Thus, face-to-face and online learning techniques must be combined. It has been reported that groups of students that complement the online learning process through virtual practices, showed a greater advance in knowledge and skills, due to the use of technologies that allow them to have unlimited access and practices, compared to students who only had a laboratory practice of approximately two pre-programmed hours per week (14).

Also, in both course, simulator-based learning is used. It has been reported that this teaching approach influences the cognitive process by establishing an environment that allows them to put into practice various strategies to solve problems (19).

It is important to recognize that in both cases, one from Latin America and one from Scandinavia, show that the use of alternative teaching resources and methods, while keeping traditional educational approaches can successfully fulfill learning outcomes.

## Perspectives and Final Notes

This manuscript described two different courses in Mexico and Denmark, where digital tools are recommended to promote student interaction and there is evidence that when both traditional classes and simulation are used in combination, student performance skills improves (13, 14).

Cognitive development is favored thanks to simulation since students have a greater opportunity to practice various strategies to solve unknowns within a common learning environment. Simulation is used as a very useful tool for students since the limitations found in a real laboratory such as the logistics limitations that exist in a physical laboratory, so these days, students quickly focus on problem solving thinking (13, 19).

To establish a relevant learning experience for students, it is necessary to innovate in the teaching-learning process in the courses. Unfortunately, a recent survey of UK schools found that roughly a considerable percentage of professors (~30%) do not offer a practical part of the courses they offer, some of the reasons are varied and included lack of time, lack of appropriate equipment and sometimes they do not have the confidence and skills to offer these practices among other reasons, so there are few microbiological activities (15, 20).

Overall, laboratory demonstrations and teaching experimental procedures for graduate students from veterinary and animal sciences can be successfully carried out as long as the learning objectives are aligned with the teaching methods and the evaluation tools. It seems that the teaching cases described in this manuscript fits with a balance between the proposed cognitive constructivism that is based on Piaget's model, which accentuates the interaction between the individual and his environment in the construction of meaningful knowledge (21) and social constructivism that was proposed by Vygotsky, which highlights the importance of learning that is achieved from the interaction between the students and the teacher (22, 23).

## Author Contributions

All authors listed have made a substantial, direct, and intellectual contribution to the work and approved it for publication.

## Conflict of Interest

The authors declare that the research was conducted in the absence of any commercial or financial relationships that could be construed as a potential conflict of interest.

## Publisher's Note

All claims expressed in this article are solely those of the authors and do not necessarily represent those of their affiliated organizations, or those of the publisher, the editors and the reviewers. Any product that may be evaluated in this article, or claim that may be made by its manufacturer, is not guaranteed or endorsed by the publisher.

## Footnotes

^1^ https://www.qschina.cn/en/university-rankings/university-subject-rankings/2021/veterinary-science
